# Disordered Gut Microbiota in Colorectal Tumor-Bearing Mice Altered Serum Metabolome Related to Fufangchangtai

**DOI:** 10.3389/fphar.2022.889181

**Published:** 2022-05-26

**Authors:** Mengmeng Cai, Ya Xiao, Zhibing Lin, Jinmiao Lu, Xiaoyu Wang, Sajid Ur Rahman, Shilan Zhu, Xiaoyu Chen, Jialin Gu, Yuzhu Ma, Zhaoguo Chen, Jiege Huo

**Affiliations:** ^1^ Affiliated Hospital of Integrated Traditional Chinese and Western Medicine, Nanjing University of Chinese Medicine, Nanjing, China; ^2^ Jiangsu Province Academy of Traditional Chinese Medicine, Nanjing, China; ^3^ Key Laboratory of Animal Parasitology of Ministry of Agriculture, Laboratory of Quality and Safety Risk Assessment for Animal Products on Biohazards (Shanghai) of Ministry of Agriculture, Shanghai Veterinary Research Institute, Chinese Academy of Agricultural Sciences, Shanghai, China; ^4^ School of Agriculture and Biology, Shanghai Jiao Tong University, Shanghai, China; ^5^ South China Agricultural University, Guangzhou, China; ^6^ College of Animal Science and Technology, Anhui Agricultural University, Hefei, China

**Keywords:** colorectal cancer, Fufangchangtai, gut microbiota, serum metabolome, traditional Chinese medicine

## Abstract

**Purpose:** This study aimed to investigate the relationship between gut microbiota (GM) and serum metabolism using antineoplastic Fufangchangtai (FFCT) as the model prescription in the treatment of colorectal cancer (CRC).

**Methods:** Tumor-bearing mice and normal mice were administered different doses of FFCT. The tumor volume of tumor-bearing mice was observed. The levels of CD4^+^ and CD8^+^ T cells in the blood, spleen, and tumor of mice were determined using a flow cytometer. The bacterial microbiota in stool samples from mice and the serum metabolomics of FFCT-treated mice and fecal microbiota transplantation mice were detected using 16s RNA sequencing and liquid chromatography–mass spectrometry (LC/MS), respectively.

**Results:** The tumor volume of mice showed no significant decrease after FFCT intervention. The levels of CD4^+^ and CD8^+^T lymphocytes showed a significant increase under the intervention of FFCT. GM of colorectal tumor-bearing mice and healthy mice were determined, and the diversity and abundance of *Firmicutes*, *Deferribacteres*, *Bacteroidetes*, and *Proteobacteria* were significantly different between the two groups. Furthermore, we found that the levels of matrine, isogingerenone B, and armillaripin were significantly decreased in tumor-bearing mice after FFCT intervention, indicating that the tumor-induced dysbiosis of gut bacteria may affect the absorption and metabolism of FFCT. Under the intervention of FFCT, serum metabolism of mice transplanted with feces from CRC patients showed less metabolites related to FFCT than that from healthy people, indicating that GM could be a single factor affecting the metabolism of FFCT. Furthermore, we found that different doses of FFCT-treated mice had higher abundance of *Roseburia*, *Turicibacter*, and *Flexispira* than that in the non-intervention control group. *Firmicutes* and *Bacteroidetes* in FFCT-treated groups showed a similar trend compared to the healthy group, indicating that FFCT might correct the intestinal microenvironment by modulating gut microbiota in colorectal tumor-bearing mice.

**Conclusion:** The dysbiosis of GM in tumor-bearing mice reduced the serum metabolites related to FFCT, and FFCT could correct the disordered GM of colorectal tumor-bearing mice to exert efficacy.

## Introduction

Colorectal cancer (CRC) is a type of cancer that affects the colon or rectum. Despite the fact that the death rate of CRC patients is steadily decreasing due to population screening and endoscopic surveillance, it is still the third highest in the world in terms of incidence and mortality (Siegel et al., 2020). Gut microbiota (GM), also known as “the new virtual metabolic organ,” plays a vital role in stimulating the digestion, producing nutrients, and promoting mucosal barrier immunity (Eckburg et al., 2005; Ramakrishna, 2013; Castellanos et al., 2018; Milosevic et al., 2019). Once GM is out of balance, it contributes to several systemic diseases, including cancer. Many pieces of evidence indicated a strong correlation between the development of CRC and GM dysbiosis (Wu et al., 2009; Zackular et al., 2013; Zhao R. S. et al., 2019). A previous study showed the alterations in the dominant and subdominant families of bacteria in CRC individuals and healthy controls (Sobhani et al., 2011). According to the “driver-passenger theory,” GM causes CRC by establishing a tumor microenvironment and inducing epithelial DNA damages and tumorigenesis (Gagniere et al., 2016). Meanwhile, GM also affects the immunity and prognosis of CRC patients by producing immunostimulatory and immunosuppressive cytokines such as IL-17A and IL-9 (Niccolai et al., 2020). Studies also showed that tumor characterized by IL-17-expressing T cells, FOXP3hi Tregs, and immunosuppressive myeloid populations were associated with worse clinical outcome (Fidelle et al., 2020).

There are numerous CRC treatments available, including radical surgery, chemotherapy, radiotherapy, targeted therapy, immunotherapy, and others. However, these treatments have limitations and cause many side effects. Therefore, traditional Chinese medicine (TCM) plays an indispensable role in the prevention of tumor recurrence and metastasis due to its effect on synergy and attenuation. Chinese decoctions such as Xiaoyaosan successfully reduced the tumor volume and tumor weight in mice with CRC xenografts and prolonged the overall survival time through a 21-day intragastric treatment (Zhang Z. et al., 2020). Herbs such as *Matricaria chamomilla* could act as a potent single component against DMH-induced CRC by modulating the Wnt signaling pathway (El Joumaa et al., 2020). Our clinical experience prescription Fufangchangtai (FFCT) has also proved to have an anti-tumor effect. FFCT is a herbal formulation consisting of *Panax ginseng* C. A. Mey (ren shen), *Actinidia chinensis* Planch (teng li gen), *Hedysarum multijugum* Maxim (huang qi), Akebiae Fructus (yu zhi zi), Coicis Semen (yi yi ren), and *Sophora flavescens* Radix (ku shen). The efficacy of FFCT was verified in our previous clinical trials, showing the anti-CRC effect by boosting the body’s immune system, improving the patient’s quality of life, and reducing side effects (Li, 2019).

As anti-tumor immunity is a part of cancer treatment, the level of immune cells can reflect the therapeutic effect of drugs. The cells involved in anti-tumor immunity mainly include T cells, NK cells, macrophages, dendritic cells (DC), etc. Among them, T-cell response is the most important host response in controlling tumor growth and development. The T-cell subsets involved in anti-tumor immunity are mainly CD4^+^ and CD8^+^ T cells. CD4^+^ T cells can enhance the function of CD8^+^ T cells by producing lymphokines, activating macrophages or other APCs, and producing tumor necrosis factor. CD8^+^T cells can secrete lymphokines and recognize the specific antigen on tumor cells so as to directly or indirectly kill tumor cells with the help of CD4^+^ T cells (Zheng et al., 2021). In our previous clinical study, we found that FFCT could increase the CD3^+^ T cell levels compared with placebo (Li, 2019).

The majority of TCM are taken orally and absorbed into the bloodstream via the gastrointestinal tract, where it exerts its efficacy. Therefore, TCM can reverse or mitigate varied compositional dysbiosis of GM associated with many diseases besides improving the pathological symptoms (Xu et al., 2017). A study showed that Berberine rescued *Fusobacterium nucleatum*-induced colorectal tumorigenesis by modulating the tumor microenvironment and blocking the activation of tumorigenesis-related pathways (Yu et al., 2015). The classical prescription, “Yi-Yi-Fu-Zi-Bai-Jiang-San” (YYFZBJS) blocked tumor initiation in treating CRC by mediating Tregs and regulating the natural GM of Apc (Min/+) mice including *Bacteroides fragilis*, *Lachnospiraceae*, and so on (Sui et al., 2020). GM also harbors many types of enzymes, allowing plenty of catalytic reactions, which are involved in the biotransformation of TCM components. Even though GM can interact complicatedly with TCM components, whether GM can influence the therapeutic effect of TCM on CRC remains unknown.

Thus, it is hypothesized that GM dysbiosis affects the serum metabolome of the human body, which is related to TCM in the treatment of CRC. To test the hypothesis, we used FFCT as a model prescription TCM to explore the correlation between GM and the metabolism of TCM in CRC treatment.

## Materials and Methods

### Preparation of Drugs

FFCT was prepared using six kinds of TCM granules, including Panax Ginseng C. A. Mey (ren shen), Actinidia Chinensis Planch (teng li gen), Hedysarum Multijugum Maxim (huang qi), Akebiae Frucyus (yu zhi zi), Coicis Semen (yi yi ren), and Sophorae Flavescentis Radix (ku shen), which were purchased from Jiangsu Hospital of Integrated Traditional Chinese and Western Medicine. The quality of FFCT was monitored using a high performance liquid chromatography (HPLC) analysis as discussed in our previous study (Wang, 2018). The formulated granules were ground into a powder and dissolved in warm distilled water before being administered to mice.

### Cells and Animals

CT26-LUC cells were provided by Shanghai Institute of Veterinary Medicine and cultured with RPMI-1640 containing 10% fetal bovine serum, 1% penicillin/streptomycin, and 4 μg/ml puromycin dihydrochloride. Reagents for cell culture were purchased from Thermo Fisher Scientific (Waltham, MA, United States). The incubator’s condition was stable at 37°C and 5% CO_2_.

All animal experiments were performed in compliance with animal welfare guidelines and approved by the Animal Ethical Committee of Shanghai Veterinary Research Institute, Chinese Academy of Agricultural Sciences (SHVRI-SZ-20200420-01, SHVRI-SZ-20200720-01). Six-week-old female BALB/C mice were purchased from Shanghai Jiesijie Company (Shanghai, China) and housed under SPF condition with 25 ± 2°C and a 12-h light/dark cycle. The mice were given free access to a standard diet and drinking water.

### Gut Microbiota Detection Experiment

To explore the difference in GM under different conditions, after 1 week of acclimatization, the mice were randomly divided into five groups: the PBS group (normal mice injected with PBS and administered with ddH_2_O, *n* = 4), the CT26 group (CRC tumor-bearing mice administered with ddH_2_O, *n* = 5), the CT26L group (CRC tumor-bearing mice administered with 0.65 mg/g FFCT, *n* = 5), the CT26M group (CRC tumor-bearing mice administered with 1.3 mg/g FFCT, *n* = 5), the CT26H group (CRC tumor-bearing mice administered with 2.6 mg/g FFCT, *n* = 5). The daily dosage for the CT26M group was obtained based on the daily dosage for patients (10 g/70 kg) clinically, according to the human–mouse transfer formula (mouse dose = human dose × 9.1) (Xu and Chen, 1992).

Before administration, 100 μl of CT26-LUC cells (1 × 10^6^/ml) were inoculated subcutaneously into the flanks of mice (CRC tumor-bearing groups), while 100 μl of PBS was inoculated subcutaneously (PBS group). About 200 μl of ddH_2_O was administered to the PBS group and CT26 group, while 200 μl of FFCT suspension was administered to the other groups via gastric gavage, and the drugs were administered for 4 weeks. Tumor growth was measured using a caliper every 3 days and tumor volumes were calculated using the following formula: volume = (length/2) × (width)^2^. Fluorescence expression in the region of interest (ROI) was used to evaluate the tumor volume based on the Small Animal 3D Multimodality Imaging System (IVIS Spectrum, PerkinElmer, Waltham, MA, United States).

Stool samples of the mice were collected 27 days after administration. Fecal pellets (2–4) were collected from each mouse using a 1.5-ml Eppendorf tube and stored at −80°C until used. Furthermore, blood was collected 24 h after the last oral administration, and serum was separated by centrifuging the blood at 3,000 rpm for 15 min at 4°C and stored at −80°C until analyzed. The stool samples collected from 4–5 mice in each group were detected.

### Fufangchangtai Serum Metabolomics Experiment

To explore the FFCT metabolites under tumor-induced gut dysbiosis condition, after 1 week of acclimatization, the mice were randomly divided into four groups: the control group (normal mice injected with PBS and administered with ddH_2_O, *n* = 6), the CT26 group (CRC tumor-bearing mice administered with ddH_2_O, *n* = 6), the CT26H group (CRC tumor-bearing mice administered with 2.6 mg/g FFCT, *n* = 6) and the CM group (normal mice injected with PBS and administered with 2.6 mg/g FFCT, *n* = 6). Before administration, 100 μl of CT26-LUC cells (1 × 10^6^/ml) were inoculated subcutaneously into the flanks of the mice (CT26 group and CT26H group), while 100 μl of PBS was inoculated subcutaneously (control group and CM group). About 200 μl of FFCT suspension was given to the CT26H group and CM group via gastric gavage, and the drug administration lasted for 4 weeks. The blood was drawn from the eyeball of the mice and allowed to clot at 4°C for 1 h. After a 15-min centrifugation at 3,000 rpm at 4°C, the supernatant was collected and stored in dry ice for blood mass spectrometry.

### Fecal Microbiota Transplantation Experiment

Fresh stool samples from CRC patients without any clinical treatment and healthy people were separately collected. The sampling procedure was approved by the Medical Ethics Committee (2105235-3) of Fudan University Shanghai Cancer Center. CRC patients including one male and three female patients were selected, and their average age was 52.6 ± 16.1. To match the numbers, healthy controls including one male and three female patients were selected with the age of 51.9 ± 4.9. The age and the gender distribution of the two groups had no significant difference (Supplementary Table S1).

Fresh stool samples (15 g per person) were mixed and suspended in 60 ml PBS. The suspension liquid was then filtered using 70-μM meshes, and the filtrate was centrifuged at 2,000 rpm for 10 min. Subsequently, after the removal of the supernatant, the remaining pellet was resuspended in 12-ml PBS (Jian et al., 2020). The mixture was used for fecal microbiota transplantation by gavage (200 μl per mouse).

Before gavage was performed, all mice were treated with a cocktail of broad-spectrum antibiotics including ampicillin (0.2 g/L), vancomycin (0.1 g/L), neomycin (0.2 g/L), and metronidazole (0.2 g/L) in drinking water for 2 weeks (Tsoi et al., 2017). All antibiotics were purchased from Shanghai Macklin Biochemical Co., Ltd. (Shanghai, China). Subsequently, the mice were randomly divided into two groups: the FMT-CA-FFCT group (mice transplanted with feces from CRC patients, *n* = 6) and the FMT-H-FFCT group (mice transplanted with feces from healthy controls, *n* = 6). These two groups were given fecal suspension from CRC patients or healthy people, respectively, for 2 weeks by gavage (twice per week), and a 200-μl high dose of FFCT (2.6 mg/g) was also given by gavage for 2 weeks at the same time. Blood samples were drawn from the eyeballs of the mice 24 h after the last oral administration and left undisturbed to cool at 4°C for 1 h. Serum was separated by centrifuging of blood at 3,000 rpm for 15 min at 4°C and stored at −80°C until analyzed.

### Flow Cytometry Analysis

The blood, spleen, and tumor tissues from different groups were separated, and the proportion of CD3^+^CD4^+^ and CD3^+^CD8^+^ T cells was analyzed using flow cytometry. The collected tumor tissues were digested with Collagenase D (1 mg/ml) and DNase I (0.5 mg/ml) and Dispase^®^ II (1.5 U/ml) in RPMI-1640 for 30 min at 37°C. The digested material was passed through a mesh (70 μM) to remove clumps and the filtrate was washed twice and then centrifuged at 400 g for 8 min at room temperature (RT). The collected blood was lysed using lyse solution, after which it was washed with PBS and then centrifuged at 700 g for 5 min. The whole spleen was placed in a cell strainer and crushed, and then passed through a 70-μm mesh to remove clumps and the filtrate was washed twice, and then centrifuged at 400 g for 8 min at RT. A total of 1×10^6^ cells were incubated for 30 min at 4°C with the following antibodies: (FITC)-CD3, (PB450)-CD4, (PE)-CD8. All the antibodies were purchased from Becton, Dickinson and Company (BD, NJ, United States). Following two washes with 1 ml of staining buffer, the cells were resuspended in 200-μl staining buffer for analysis on a CytoFLEX flow cytometer (Beckman Coulter Life Sciences, Brea, CA, United States).

### Gut Microbiota Analysis

The OMEGA Soil DNA Kit (D5625-01) (Omega Bio-Tek, Norcross, GA, United States) was used to extract the DNA from fecal samples. The quantity and quality of extracted DNAs were measured using a NanoDrop ND-1000 spectrophotometer (Thermo Fisher Scientific, Waltham, MA, United States) and agarose gel electrophoresis, respectively.

Sequencing of the 16S rRNA genes was performed using the Illumina MiSeq platform. Universal 16S primers (338F/806R) with a sample-specific 12 bp barcode were used to amplify the hypervariable V3-V4 region of bacterial 16S rRNA genes. PCR was performed using a thermal cycler Model C1000 (Bio-Rad, Richmond, CA, United States) (Church et al., 2020). The 16s RNA sequencing was performed by Personal Bio (Shanghai, China).

Microbiome bioinformatics were processed and analyzed with the QIIME2 (2019.4) software package (Bolyen et al., 2018). Briefly, Raw sequence data were demultiplexed using the demux plugin following by primers cutting with cutadapt plugin (Martin, 2011). Sequencing reads were quality-filtered, denoised, merged, and chimera removed using the DADA2 plugin (Callahan et al., 2016). Non-singleton amplicon sequence variants (ASVs) were aligned with mafft and used to construct a phylogeny with FastTree-2 (Katoh et al., 2002; Price et al., 2009). Alpha and beta diversity metrics were estimated using the diversity plugin. Taxonomy was assigned to ASVs using the classify-sklearn naïve Bayes taxonomy classifier in feature-classifier plugin against the SILVA Release 132 Database (Kõljalg et al., 2013; Bokulich et al., 2018). QIIME-generated ASV tables were used for downstream statistical analysis.

### Serum Metabolomics Analysis

Metabolites are stable in serum and can be quantified, which presents an opportunity in monitoring disease status and exploring biomarkers to predict the efficacy of anticancer therapies (Tian et al., 2018). The serum metabolomics of two groups (CM, CT26H) were detected. The blood was drawn from the eyeball of mice and then stored at 4°C for 1 h. After 15 min of centrifugation at 3,000 rpm at 4°C, the supernatant was collected and stored in dry ice for blood mass spectrometry. Liquid chromatography–mass spectrometry (LC/MS) analysis of the blood was entrusted to Luming Bio company, (Shanghai, China).

A measure of 100 μl of serum from each sample was vortexed with a 10-μl internal standard (0.3 mg/mL L-2-chlorophenylalanine or 0.01 mg/ml Lyso PC 17:0 dissolved in methanol) for 10 s. The mixtures were precipitated by 300-μl mixtures of methanol and acetonitrile (2/1, v/v) and then ultra-sonicated on ice for 10 min, then at −20°C for 30 min. After 10 min of centrifugation (13,000 rpm, 4°C), 300 μl of supernatant from each sample was collected and dried. The dried supernatant was resolved in 400 μl of 20% methanol and then placed at –20°C for 2 h. Then, 150 μl of supernatant per sample was filtered through a 0.22-μM microfilter and transferred into LC sampling vial for LC/MS analysis. Additionally, a quality control (QC) sample was created by mixing an aliquot of equal volume of each sample.

A Dionex UltiMate 3000 RS UHPLC system (Thermo Fisher Scientific, Waltham, MA, United States) coupled with Q-Exactive quadrupole-Orbitrap mass spectrometer (Thermo Fisher Scientific, Waltham, MA, United States) was used to analyze the metabolic profiling in both ESI-positive and ESI-negative ion modes. A quantity of 2 μl of the prepared sample was injected into the ACQUITY UPLC HSS T3 column (1.8 μM, 2.1 × 100 mm). All samples were eluted using a linear gradient from 100% mobile phase A (0.1% formic acid in water) to 100% mobile phase B (0.1% formic acid in acetonitrile) under the condition that the flow rate was 350 μl/min and the column temperature was 45°C. Linear gradients: 0 min, 5% B; 2 min, 5% B; 4 min, 25% B; 8 min, 50% B; 10 min, 80% B; 14 min, 100% B; 15 min, 100% B; 15.1 min, 5% B; and 16 min, 5% B. The electrospray ionization (ESI) source operating in positive and negative mode (Water, Milford, MA, United States) was used for the mass spectrometry analysis. Parameters of mass spectrometry were as follows: Capillary temperature was set at 320°C, while the aux gas heater temperature was set at 350°C. Sheath gas flow rate was 35 Arb and aux gas flow rate was 8 Arb. The san range was from 100 to 1,000 m/z.

The raw data were processed by the Progenesis QI v2.3 software (Nonlinear Dynamics, Newcastle, United Kingdom) for baseline filtering, peak recognition, peak alignment, and retention time correction. The Human Metabolome Database (HMDB), LIPID MAPS (v2.3), and METLIN database, and a self-built database were used to identify the compounds.

### Statistical Analysis

Statistical analysis was performed using Version 22 of SPSS for Windows, and differences were considered significant at a *p*-value < 0.05. Distributions of variables were examined by Shapiro–Wilk Test according to our sample size, and appropriate tests were applied for further analysis. The tumor volume and percentage of immune cells in different groups were analyzed with analysis of variance (ANOVA). Means from the data, together with estimates of the standard error of the mean and pairwise comparisons (Tukey’s or Games-Howell test), were obtained. Clustering of gut microbial communities among different groups was analyzed with Adonis. The alpha diversity analysis was conducted by Dunn’s test, while the beta diversity analysis, using the Permanova test. Pairwise comparison in two abnormal distribution groups was analyzed with Mann–Whitney U test. In the analysis of serum mass spectrometry data, the unsupervised principal component analysis (PCA) was used to observe the distribution of samples and the stability of the analysis process, and then the supervised orthogonal partial least squares discrimination analysis (OPLS-DA) was used to describe the differences between the groups. The contribution of each variable to the population was ranked according to the variable importance of projection value (VIP value) obtained from the OPLS-DA model, and the significance of differential metabolites between the groups was verified by a *t* test and fold change analysis. The Pearson correlation coefficient was used to measure the linear correlation between the two groups. It was considered that the difference was statistically significant when VIP >1.0 and *p* < 0.05.

## Results

### Fufangchangtai Regulated the Levels of CD4^+^ and CD8^+^ T Cells

**FIGURE 1:**
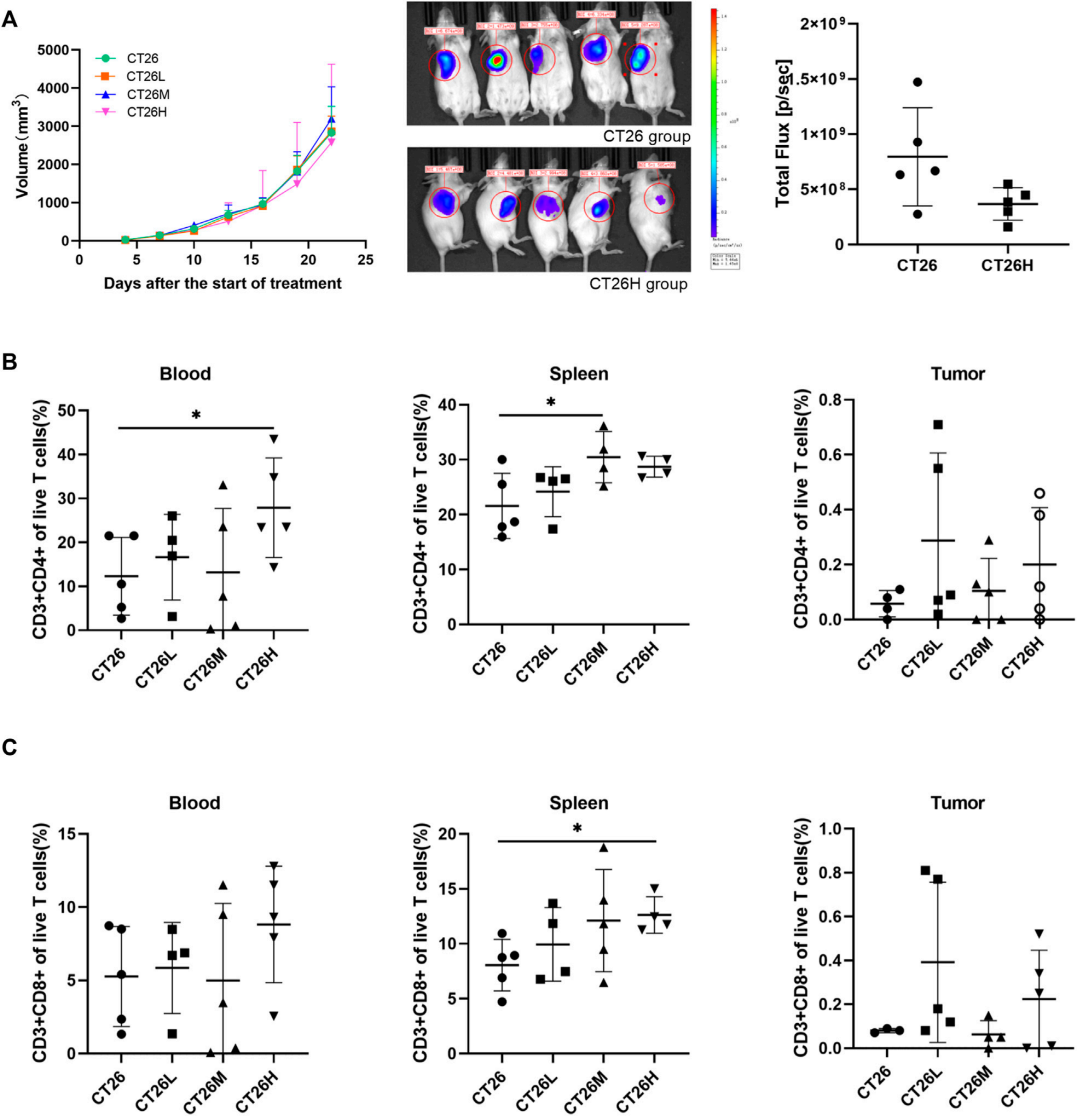
Tumor volume and percentage of lymphocytes among the CT26 group and FFCT-treated groups. **(A)** Tumor volume among the CT26 group and FFCT-treated groups (*p* > 0.05). Percentage of CD3^+^CD4^+^ **(B)** and CD3^+^CD8^+^ T cells **(C)** in the blood, spleen, and tumor tissue of the CT26 tumor-bearing mice treated with FFCT. * represents *p* < 0.05.

To analyze the curative effect of FFCT during CRC treatment, we tested the tumor volume and assessed the T cells (CD4^+^CD8^+^) in the blood, spleen, and tumor using a CRC mouse model. As shown in Figure 1A, FFTC treatment had no significant influence on the tumor volume in each group (*p* > 0.05). Furthermore, we analyzed the levels of immune cells (CD3^+^CD4^+^ and CD3^+^CD8^+^ T cell) using flow cytometry. Gating strategies are shown in Supplementary Figure S1. The results showed that CD3^+^CD4^+^ T cells from the spleen and blood in the FFCT-treated groups were significantly increased compared with the CT26 group, but there was no significant difference in the tumor volume between the two groups (Figure 1B). Compared with the CT26 group, the CD3^+^CD8^+^ T cells from the spleen in the CT26H group were increased, but there was no significant difference in the blood and tumor between the two groups (Figure 1C). These results suggested that FFCT might enhance the body immunity by increasing the levels of CD3^+^CD8^+^ and CD3^+^CD4^+^ T cells in the blood and spleen.

### Fufangchangtai Changed the Gut Microbiota in CRC Tumor-Bearing Mice Model

**FIGURE 2:**
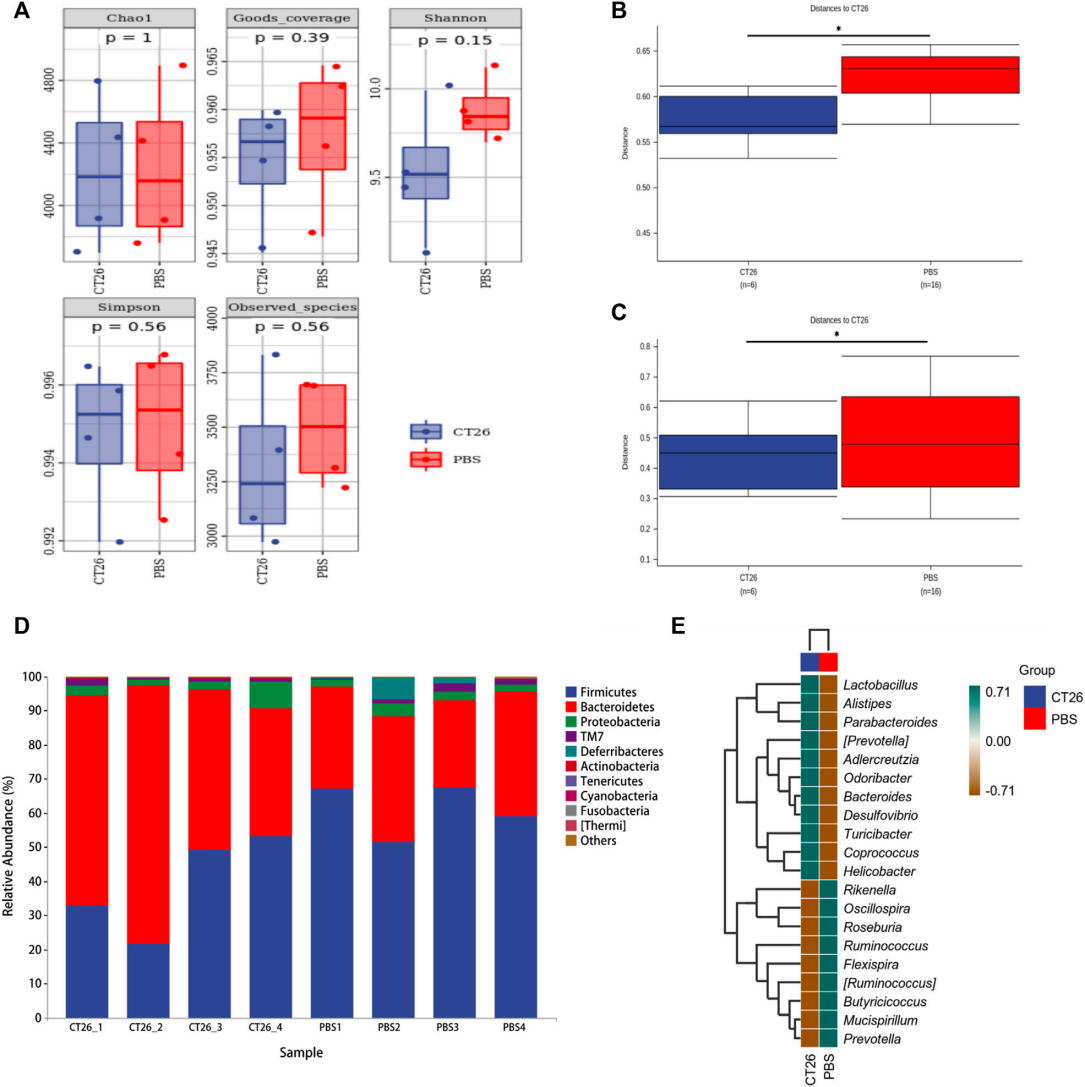
GM differences between the CT26 group and the PBS group. **(A)** Diversity index analysis (Chao1, Good’s coverage, Shannon, Simpson, and Observed species) between the CT26 group and the PBS group. **(B)** Differences in unweighted UniFrac distance between the CT26 group and the PBS group. **(C)** Differences in weighted UniFrac distance between the CT26 group and the PBS group. **(D)** Total gut bacterial relative abundance at the taxonomic rank of phylum. **(E)** Heat map of species composition of the gut microbiota in the CT26 group and the PBS group at the genus level. * represents *p* < 0.05.

The gut microbiome of different groups (PBS, CT26, CT26L, CT26M, CT26H) was analyzed using 16s RNA sequencing. The alpha diversity analysis showed that there were no significant differences between the CT26 and PBS group (Dunn’s test, *p* > 0.05) (Figure 2A). However, beta diversity analysis using both unweighted and weighted UniFrac distances indicated that the PBS group had higher dissimilarities among gut microbial communities than the CT26 group (Permanova, *p* < 0.05; Figures 2B,C). The rarefaction curves were close to the saturation platform at the sequencing depth of 10,000, indicating that the data were sufficient to reflect the GM information about all the samples (Supplementary Figure S2A). The Venn diagram (Supplementary Figure S2B) revealed 1,395 common OTUs across all the five groups. The results showed a significant difference in GM between the tumor-bearing mice and the healthy mice. The principal coordinates analysis (PCoA) revealed the similarity in species abundance composition of two samples in the corresponding dimensions by analyzing the projection distance of the samples on the coordinate axis. Bray and Curtis distance here was used to characterize the community differences between the samples. As for the CT26 group and the PBS group, the PCoA diagram (Supplementary Figure S3A) suggested significantly distinct clustering of gut microbial communities between the two groups (Adonis, *p* < 0.05). The rank abundance curve consists of broken lines, and each broken line represents a group. The length of the broken line on the horizontal axis reflects the number of OTU in the specific abundance. The smoother the broken line, the smaller is difference of the OTU diversity in the community. As for the CT26 group and the PBS group, the abundance grade curve (Supplementary Figure S3B) demonstrated that the PBS group has higher community similarity than the CT26 group.

The data in Figure 2D showed that the composition of the GM in the two groups (CT26 group and PBS group) was mainly characterized by the *Firmicutes*, *Bacteroidetes*, *Proteobacteria*, *TM7*, and *Deferribacteres*. A marked difference between the two groups existed on the distribution of phyla. The abundance of *Firmicutes* and *Deferribacteres* in PBS group was higher than that in the CT26 group (*p* < 0.05), while the abundance of *Bacteroidetes* and *Actinobacteria* was lower (*p* < 0.05). As shown in Figure 2E, species composition in the two groups at the genus level was quite different as well. Species with high expression in the CT26 group were *Lactobacillus*, *Alistipes*, *Parabacteroides*, (*Prevotella*), *Adlercreutzia*, *Odoribacter*, *Bacteroides*, *Desulfovibrio*, *Turicibacter*, *Coprococcus*, and *Helicobacter*. The species with low expression in the CT26 group were *Rikenella*, *Oscillospira*, *Roseburia*, *Ruminococcus*, *Flexispira*, (*Ruminococcus*), *Butyricicoccus*, *Mucispirillum*, and *Prevotella*.

### Tumor-Induced Gut Microbiota Dysbiosis Could Affect Fufangchangtai Metabolites

**FIGURE 3:**
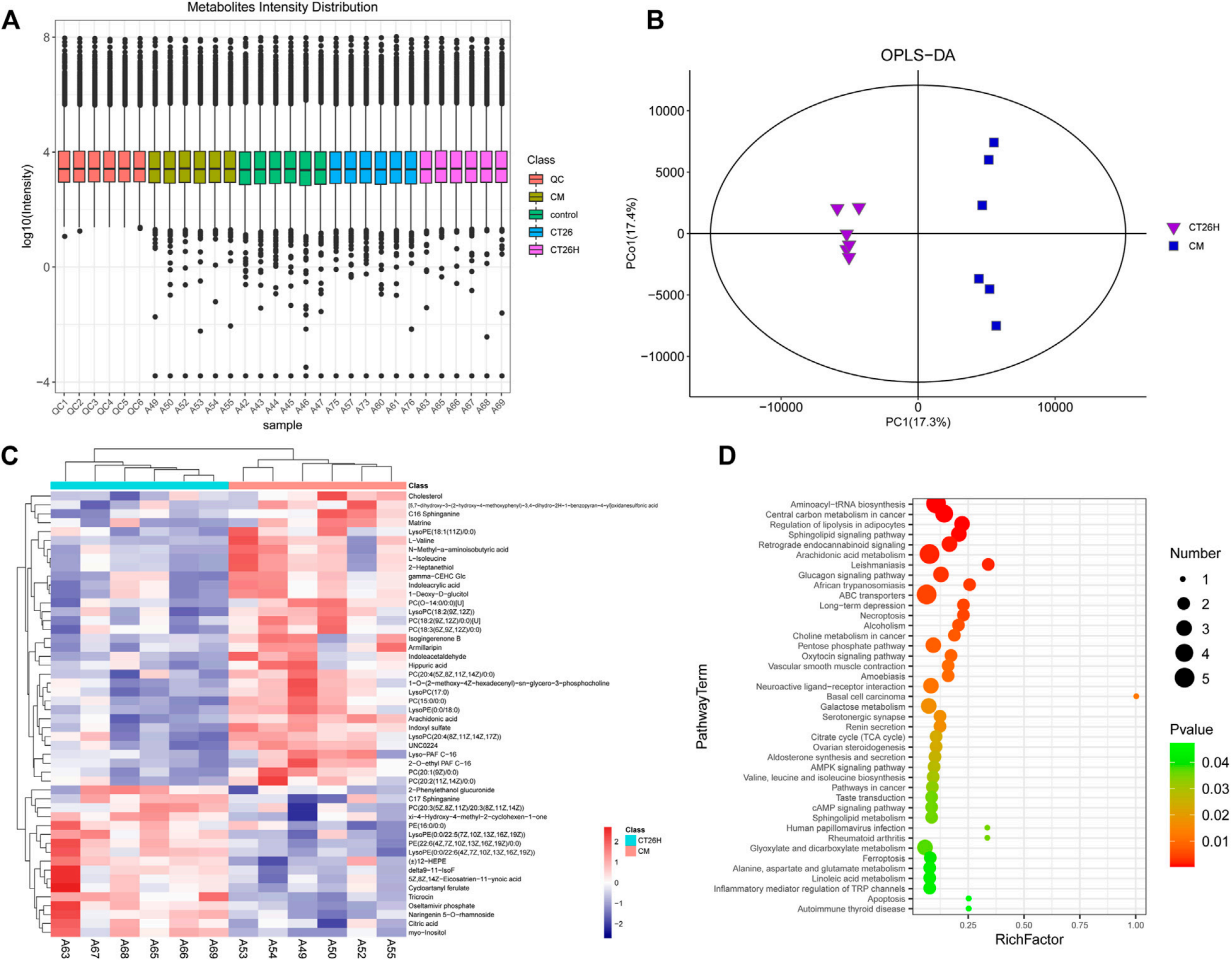
Mass spectrum of serum metabolism in the CT26H and CM groups. **(A)** Metabolites Intensity Distribution box plot of all samples. **(B)** OPLS-DA score plot of the CT26H and CM groups. **(C)** Heat map of the top 50 differential metabolites between the CT26H and CM groups. **(D)** KEGG pathways enriched by significantly different metabolites between the CT26H and CM groups (*p* < 0.05).

A high dose of FFCT (2.6 mg/g) was used in tumor-bearing mice (CT26H group) and healthy mice (CM group) for the comparison of serum metabolism between the two groups. In the PCA plot (Supplementary Figure S4), the quality control (QC) samples were closely gathered in the center of the scoring chat, showing the good stability of the instrument detection in this experiment. Results of OPLS-DA plot and the top 50 differential metabolites between the CT26 and CM group are shown in Supplementary Figure S5, indicating the metabolism of the CT26 group before treatment. In the Metabolites Intensity Distribution box plot (Figure 3A), the median line of each group was on a horizontal line, indicating that the samples were relatively stable. In addition, the dots represented the degree of dispersion, showing a relatively low dispersion in the CM group and CT26 group. The OPLS-DA plot can reflect the variability between the groups and within the groups, as well as the general distribution trend among different samples. OPLS-DA showed a clear separation between the CT26H and CM group, which indicated that the two groups indeed harbored significantly different bacterial microbiota (Figure 3B).

Overall, 243 differential metabolites were found between the CT26H group and the CM group. The top 50 differential metabolites and KEGG pathways enriched are shown in Figures 3C,D. Serum metabolites including cholesterol, matrine, isogingerenone B, armillaripin, hippuric acid, arachidonic acid, and amino acids (L-valine, N-methyl-a-aminoisobutytric acid, L-isoleucine) were mainly enriched in the CM group. Combined with network retrieval, matrine, isogingerenone B, and armillaripin are mainly related with TCM, indicating that the presence of tumor could affect the metabolism of FFCT (Figure 3C). The data in Figure 3D show that these differential metabolites were involved in multiple pathways including aminoacyl-tRNA biosynthesis, central carbon metabolism in cancer, regulation of lipolysis in adipocytes, sphingolipid signaling pathway, arachidonic acid metabolism, choline metabolism in cancer and ferroptosis, etc.

### Disordered Gut Microbiota of CRC Patients Affected Fufangchangtai Metabolites

**FIGURE 4:**
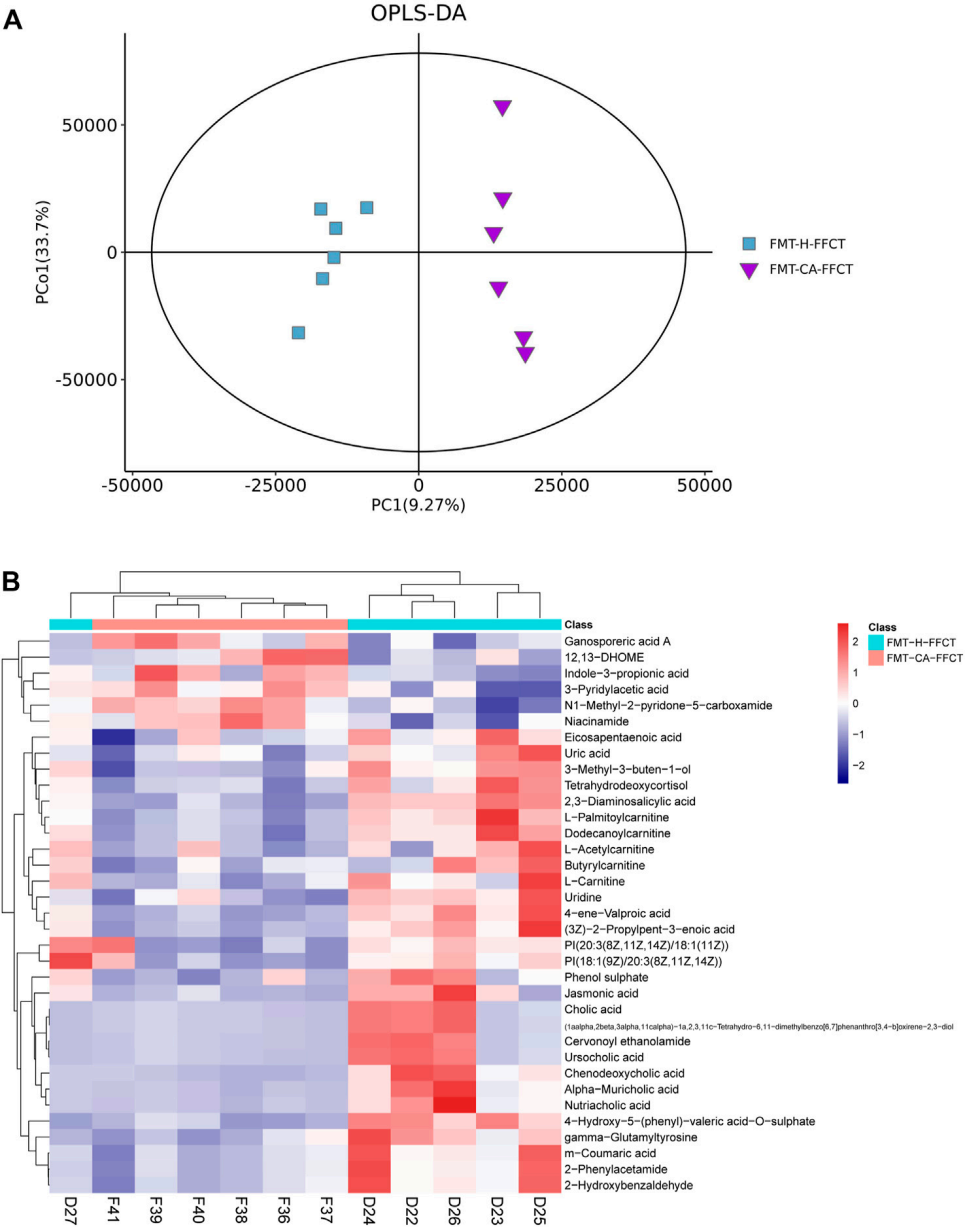
Mass spectrum of serum metabolism in FMT mice. **(A)** OPLS-DA score plot of the FMT-CA-FFCT and FMT-H-FFCT groups. **(B)** Heat map of the top 50 different metabolites between the FMT-CA-FFCT and FMT-H-FFCT groups (*p* < 0.05).

The above results showed that disordered GM in colorectal tumor-bearing mice altered the serum metabolome related to FFCT. However, the gut microbiota, or the tumor, that played the key role in the changes in the serum metabolome was unclear. A high dose of FFCT (2.6 mg/g) was used in the FMT-CA group (FMT-CA-FFCT group) and the FMT-H mice (FMT-H-FFCT group) for the comparison of serum metabolism between the two groups excluding tumor factors. The PCA plot (Supplementary Figure S6A) and Metabolites Intensity Distribution box (Supplementary Figure S6B) indicate that the samples were relatively stable. As the OPLS-DA plot shows (Figure 4A), samples of the two groups (FMT-CA-FFCT and FMT-H-FFCT) were separated clearly, showing that there was certain difference between the two groups. There were 35 different metabolites between the FMT-CA-FFCT group and the FMT-H-FFCT group. The top 50 differential metabolites between the two groups were identified as listed in the hierarchical clustering heat map (*t*-test, *p* < 0.05; Figure 4B). Serum metabolites including uric acid, jasmonic acid, m-coumaric acid, cholic acid, ursocholic acid, chenodeoxycholic acid, gamma-glutamyltyrosine, and some kinds of amino acids (butyrylcarnitine, L-palmitoylcarnitine) were higher in the FMT-H-FFCT group than in the FMT-CA-FFCT group. Combined with network retrieval, jasmonic acid and m-coumaric acid are related to TCM, indicating that gut microbiota could affect the metabolism of FFCT as a single factor.

### Fufangchangtai Ameliorated the Gut Microbiota in Tumor-Bearing Mice

**FIGURE 5:**
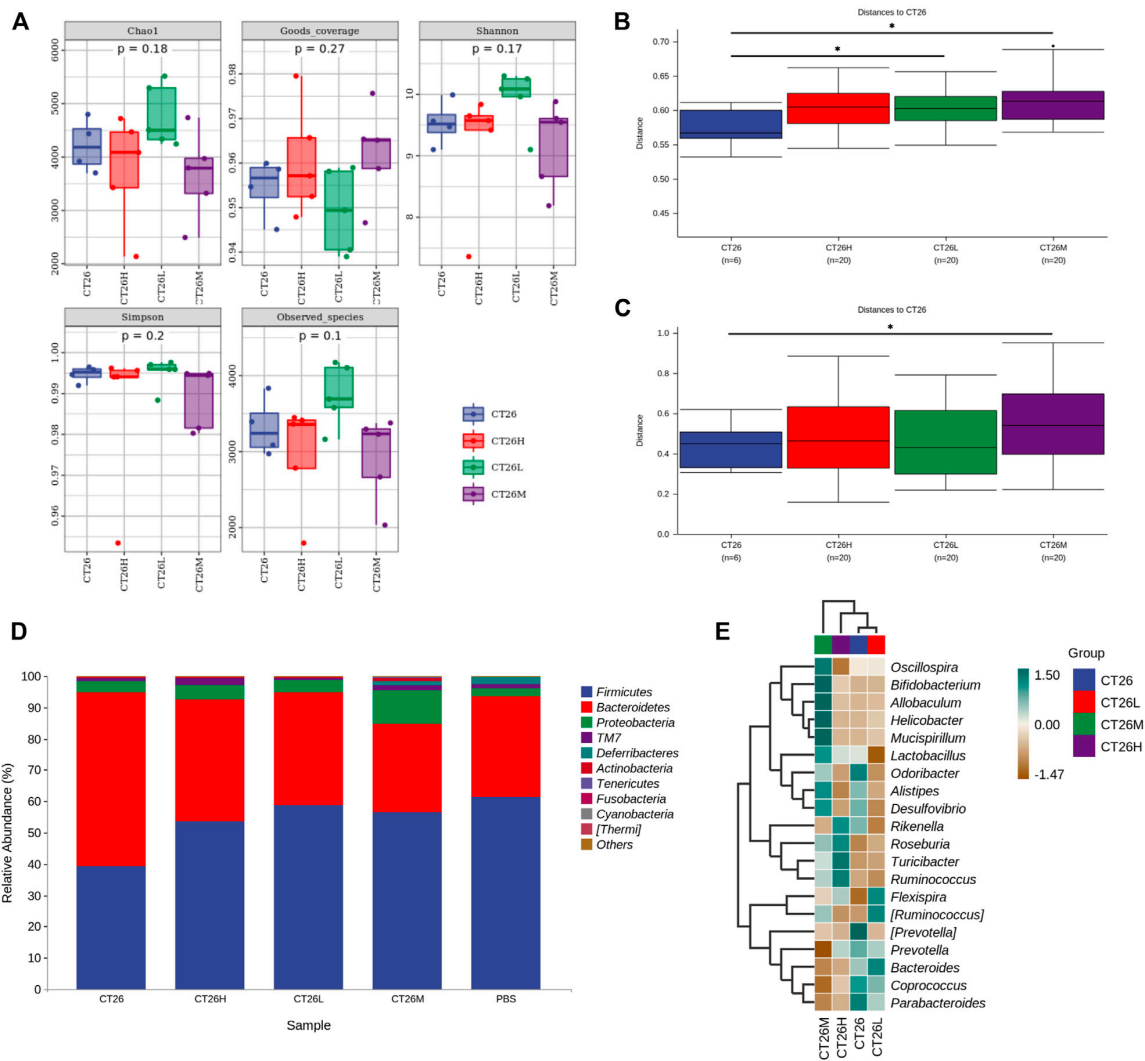
GM differences between the CT26 group and the FFCT-treated groups. **(A)** Diversity index analysis (Chao1, Good’s coverage, Shannon, Simpson, and Observed species) between the CT26 group and the FFCT-treated groups. **(B)** Differences in unweighted UniFrac distance between the CT26 group and the FFCT-treated groups. **(C)** Differences in weighted UniFrac distance between the CT26 group and the FFCT-treated groups. **(D)** Total gut bacterial relative abundance at the taxonomic rank of phylum. **(E)** Heat map of species composition of the gut microbiota in the CT26 group and the FFCT-treated groups at the genus level. * represents *p* < 0.05.

As shown in the aforementioned results, GM and serum metabolism of FFCT between tumor-bearing group and healthy group were quite different. The dysbiosis of GM in tumor-bearing mice was corresponding to less serum metabolites that related to FFCT. Next, 16S rRNA gene sequencing was used to detect the effect of different doses of FFCT on the GM of tumor-bearing mice. As expected, compared with the CRC tumor-bearing group (CT26), FFCT-treated groups (CT26L, CT26M, CT26H) had different a composition of the GM. The PCoA diagram (Supplementary Figure S7A) showed that the species abundance composition of the CT26L and CT26M groups was different from the CT26 group (Adonis, *p* < 0.05). The abundance grade curve (Supplementary Figure S7B) showed that the FFCT-treated groups had higher community similarity than the CT26 group. The alpha diversity analysis (Figure 5A) showed that there were no significant differences in Chao1, Good’s coverage, Shannon, Simpson, and Observed species indices between the CT26 group and the FFCT-treated groups (Dunn’s test, *p* > 0.05). However, beta diversity analysis using both unweighted and weighted UniFrac distances indicated that the medium dose FFCT-treated groups had higher dissimilarities among gut microbial communities than the CT26 group (Permanova, *p* < 0.05; Figures 5B,C).

The taxonomy in Figure 5D shows that the composition of GM in all the five groups was mainly characterized by *Firmicutes*, *Bacteroidetes*, *Proteobacteria*, *TM7*, and *Deferribacteres*. In the distribution of phyla, FFCT-treated groups had higher abundance of *Firmicutes* than the tumor-bearing group, which showed a similar trend with the healthy group. Moreover, with the increase in FFCT concentration, the abundance of *Firmicutes* was decreased. On the contrary, FFCT-treated groups had lower abundance of *Bacteroidetes* than the tumor-bearing group, which also showed a similar trend with the PBS group (Mann–Whitney U, *p* > 0.05). The CT26M group had the least abundance of *Bacteroidetes* and the most abundance of *Proteobacteria* and *Actinobacteria*.

As shown in Figure 5E, the following species had a high expression in the CT26 group but a low expression in the FFCT-treated groups: *Odoribacter*, (*Prevotella*), *Prevotella*, *Coprococcus*, and *Parabacteroides*. On the contrary, species like *Roseburia*, *Turicibacter*, and *Flexispira* in the CT26 group were much less expressed than in the FFCT-treated groups. Among these species, *Roseburia* and *Turicibacter* increased with an increase in FFCT concentration.

## Discussion

Nowadays, because of its multi-target effect and low toxicity, Chinese medicine has become an essential component in the adjuvant treatment of tumors. However, the mechanism of the therapeutic effect is still not understood. Studies showed that emodin could induce the cancer cell apoptosis via endoplasmic reticulum stress-dependent events, which produced reactive oxygen species (ROS) and regulated the signaling pathways (Zhang Y. et al., 2020). Qinrui Han *et al.* showed that bufarenogin induced intrinsic apoptosis through the cooperation of Bax and adenine nucleotide translocator (Han et al., 2021). Xiao-Qin Zhu *et al.* treated the CRC HCT-116 xenograft mouse model with Qingjie Fuzheng granules and found that this compound suppressed tumor cell proliferation through inhibiting the SHh pathway (Zhu et al., 2020). Yang Li *et al.* performed research on Gegen Qinlian decoction. They tested the immune cells, the cytokines, and the intestinal mucosa tight protein and showed that Gegen Qinlian decoction could enhance immunity and protect the intestinal barrier function by decreasing the relative abundance of *Megamonas* and *Veillonella* and increasing the relative abundance of *Bacteroides*, *Akkermansia*, and *Prevotella* (Li et al., 2020). As Yang’s study showed, GM had become a considerable factor in the development and treatment of CRC (Yang and Yu, 2018). Jiyoung Ahn *et al.* tested the community of GM in a study of 47 CRC subjects and 94 control subjects. The authors found that the CRC subjects had a decreased overall microbial community diversity, showing the potentially modifiable nature of the GM and the difference in the GM between CRC patients and healthy people (Ahn et al., 2013). GM is an important part of intestinal mucosal barrier and intestinal immunity. Furthermore, GM can influence physiological and pathological processes of the whole body, including the bioavailability and metabolism of macronutrients and micronutrients as well as metabolites. The vast majority of Chinese medicine are absorbed through intestinal tract to achieve the therapeutic effect, so there is the hypothesis that the GM could affect the absorption and metabolism of Chinese medicine.

In this study, we selected Fufangchangtai (FFCT) as the model prescription drug. The previous multicenter, randomized, double-blind clinical trial found that FFCT improved life quality, improved immune function, and increased the median survival time of CRC patients (Li, 2019). However, the mechanism of its anti-tumor effect is unclear; thus, we chose FFCT as the research object.

Instead of the AOM/DSS induction mice model, the subcutaneous transplantation mice model was used as the CRC tumor-bearing mice model. The AOM/DSS induction mice model is developed by drug administration through the gastrointestinal tract, which not only affects the experimental results of the GM but also has the disadvantages of long modeling cycle, unstable effect, and high mortality. The subcutaneous transplantation mice model is more intuitive and makes the experimental results stable and reliable.

By measuring the tumor volume and fluorescence expression in the ROI, the statistical results showed that FFCT could inhibit the growth of tumor to a certain extent. Perhaps expanding the sample size would help to obtain significant statistical results. At the same time, immune cells including CD4^+^ and CD8^+^ T lymphocytes were detected and showed an obvious proliferation under the intervention of FFCT, which was consistent with the results of FFCT clinical research. Studies showed that CD4^+^ T lymphocytes could take on activities in the immune response against tumor by secreting cytokines and activating CD8^+^ T lymphocytes (Zheng et al., 2021). It has been shown that high expression of CD8^+^ T cells linked to a good prognosis in multiple tumors, including colorectal cancer, esophageal cancer, and gastric cancer (Lee et al., 2018; Zhao Y. et al., 2019; Hao et al., 2020). Therefore, our results showed that FFCT exerted anti-tumor effect by enhancing the body’s immune responses.

The GM in fecal samples of CRC mice and healthy mice showed that the abundance of *Firmicutes* decreased and *Bacteroidetes* increased in tumor-bearing mice, which were consistent with findings from clinical analysis of CRC patients (Abreu and Peek, 2014). Our study showed that *Coprococcus*, *Helicobacter*, *Desulfovibrio*, etc. had a higher expression in the CT26 group. *Coprococcus*, which is linked to butyrate production and SCFA metabolism (Lin et al., 2021; Liu et al., 2021), was found to be more prevalent in tumor-bearing mice. Sarah Vascellari’s study proposed that the high expression of *Coprococcus* was associated with the development of gut inflammatory environment and gastrointestinal dysfunctions (Vascellari et al., 2021) are the pathological factors of CRC. *Helicobacter*, especially *helicobacter hepaticus*, can lead to an increase in oxidative phosphorylation that may increase DNA-damaging free radicals (Wang et al., 2021). Verena Friedrich colonized a spontaneous fatal colitis transgenic mouse with *helicobacter hepaticus* and discovered that the disease progressed quickly, indicating that this type of bacteria was a disease driver in the colitis model and was not conducive to maintaining intestinal homeostasis (Friedrich et al., 2021). *Desulfovibrio* is considered the virulent bacterium that plays a role in destroying colonic mucosa and inducing intestinal inflammation and bacterial translocation (Yan et al., 2021). Because of the significant difference in the GM between the CRC tumor-bearing mice (CT26 group) and the healthy mice (PBS group), further study was allowed to be carried out in the tumor-bearing model mice.

We detected the serum metabolism of tumor-bearing mice and healthy mice after administration of FFCT to explore whether the dysbiosis of GM under the burden of tumor had an impact on serum metabolism that related to TCM. Metabolites that related to FFCT, such as matrine, isogingerenone B, and armillaripin, were higher in the CM group than in the CT26H group. Matrine is an alkaloid compound isolated from *Radix sophorae flavescentis*, one of the components of FFCT. Many studies showed that matrine has a significant anti-tumor effect (Zhang et al., 2019; Cheng et al., 2020; Gu et al., 2020). Yun Cheng *et al.* treated CRC cells with different concentrations of matrine and found out that matrine could suppress proliferation, migration, and invasion and induce apoptosis of CRC cells via the miR-10b/PTEN pathway (Cheng et al., 2020). Yawei Zhang *et al.* showed that matrine could inhibit SW480 cell survival by activating MIEF1-related mitochondrial division via the LATS2-Hippo pathway (Zhang et al., 2019). Chen Gu *et al.* found that matrine could block circSLC7A6 exosome secretion from cancer-associated fibroblasts. In addition, circSLC7A6 acted both as a promoter for CRC cell proliferation and inhibitor for apoptosis (Gu et al., 2020). Isogingerenone B and armillaripin can be detected, but not quantified in herbs. Studies showed that isogingerenone B was most likely formed from Curcumine (Endo et al., 1990). Curcumine could inhibit human colon cancer cellular growth through cell cycle arrest at the G2/M and G1 phases, as well as stimulated apoptosis by interacting with multiple molecular targets (Pricci et al., 2020). Armillaripin is a kind of proto IRU type sesquiterpenol aromatic ester that is isolated from the mycelium of *Armillaria mellea* (Yang et al., 1990). And sesquiterpenes have a wide range of biological activities, such as anti-tumor, anti-bacterial, anti-inflammatory, antiviral, and so on (Lu Li et al., 2018). The abovementioned three main different metabolites demonstrated that FFCT had a significant effect on cancer treatment. According to the findings, groups with healthy microbiota had more serum metabolites related to FFCT. To confirm that whether GM or tumor itself played the key role in the change of serum metabolome, we detected the serum metabolism of mice transplanted with feces from CRC patients and healthy people, respectively. Metabolites that related to FFCT-like jasmonic acid and m−coumaric acid were much less in the FMT-CA-FFCT group than in the FMT-H-FFCT group, indicating that GM could affect the metabolism of FFCT as a single factor. When combined with the findings of GM experiment, the conclusion could be summarized as follows: a relatively healthy intestinal flora and microenvironment was conducive to drug absorption and metabolism into the blood, resulting in a better therapeutic effect.

Further analysis of metabolic pathways showed that the therapeutic effect of FFCT might be predominantly relevant to the ability of regulating aminoacyl-tRNA biosynthesis, central carbon metabolism in cancer, regulation of lipolysis in adipocytes, and arachidonic acid metabolism. The aminoacyl-tRNA synthetases (ARSs) are an essential and universally distributed family of enzymes that plays a critical role in protein synthesis (Rubio Gomez and Ibba, 2020). Certain ARSs, such as isoleucyl-tRNA synthetase 2 (IARS2) and lysyl-tRNA synthetase (KRS), have been shown to promote colon cancer development. KRS was involved in colon cancer metastasis by inducing M2 macrophage polarization. Soluble factors secreted by M2 macrophages not only induced the activation of intracellular signals in cancer cells, but also activated the adjacent cancer-associated fibroblast, which ultimately remodeled the tumor microenvironment and exacerbated cancer metastasis (Nam et al., 2018). Central carbon metabolism traditionally includes the glycolysis pathway (EMP), pentose phosphate pathway (PPP), and tricarboxylic acid cycle (TCA), and it is the main source of energy required by organisms and provides precursors to other metabolisms in the body. Lipolysis in adipocytes is a key feature of some chronic diseases such as cancer-associated cachexia (CAC) (Yang et al., 2021). Thorhallur Agustsson *et al.*, made a point that although loss of adipose tissue may be less harmful than muscle loss in cancer cachexia, the former seems to precede the latter. Maybe early regulation of lipolysis in adipocytes could slow down or prevent the progressive wasting among cancer patients (Agustsson et al., 2007). The metabolism of arachidonic acid through cyclooxygenase (COX), lipoxygenase (LOX), and cytochrome P-450 epoxygenase (EPOX) pathways leads to the generation of various biologically active eicosanoids, and inhibition of these pathways has generally been shown to inhibit tumor growth/progression (Cathcart et al., 2011). These findings suggested that FFCT could not only regulate pathways related to the occurrence, growth, invasion, and metastasis of tumor, but also involve in substance synthesis and energy supplementation in serum metabolism.

Simultaneously, FFCT was administered to CRC tumor-bearing mice in order to observe GM changes in response to TCM intervention. It was found that the composition of GM in tumor-bearing mice differed significantly before and after FFCT administration. Many pieces of evidence demonstrated that *Firmicutes* was a phylum with documented anti-tumorigenic effects (Bader et al., 2018). *Bacteroidetes*, on the contrary, could drive DNA damages in colon epithelial cells as a potential “driver” of CRC (Tjalsma et al., 2012). As the abundance of *Firmicutes* was higher and the abundance of *Bacteroidetes* was lower in the FFCT-treated groups, and *Firmicutes*/*Bacteroidetes* was generally regarded as having a significant relevance in the signaling of GM status (Ley et al., 2006). The results indicated that the imbalance of GM in tumor-bearing mice had been regulated by FFCT. The intervention of FFCT increased the abundance of *Turicibacter* and *Roseburia*, and as the FFCT concentration increased, the abundance of these two species increased. *Turicibacter* is involved in fermentation metabolism, and its main metabolite is lactic acid, which has the function of regulating the muscle and anti-fatigue. *Roseburia* can ferment a variety of carbohydrates, increase the content of butyric acid in the intestine, and prevent or treat obesity-related diseases. Studies showed that *Turicibacter* and *Roseburia* had a negative association with intestinal dystrophy (Zhong et al., 2022). Combining the research findings, it is concluded that FFCT altered the structure of GM while maintaining enteral nutrition. However, the specific intestinal protection mechanism needs to be studied further.

This study has limitations. Although there were immunological changes, FFCT showed no direct inhibitory effect on tumor. The combination of FFCT and immunotherapy could be considered to give full play to the anti-tumor effect. Gut microbiota and serum metabolism is a dynamic and complex process. Further studies on the interaction between specific active components of FFCT and targeted gut bacteria may shed more light on these mechanisms.

## Conclusion

It was pointed out that GM dysbiosis under CRC burden blocked the absorption and metabolism of FFCT, indicating that a healthier intestinal microenvironment was conducive for better efficacy of FFCT. Moreover, FFCT could correct the imbalance in GM of CRC individuals. This study provided a new perspective on the therapeutic effect of FFCT by combining gut microbiota and serum metabolism, as well as a foundation for improving the GM status of CRC patients in clinical treatment, with the goal of achieving a better curative effect.

## Data Availability

The datasets presented in this study can be found in online repositories. The names of the repository/repositories and accession number(s) can be found below: https://www.ncbi.nlm.nih.gov/bioproject/PRJNA815928

## Abbreviations

ANOVA, analysis of variance; ARSs, aminoacyl-tRNA synthetases; ASVs, amplicon sequence variants; CAC, cancer-associated cachexia; COX, cyclooxygenase; CRC, colorectal cancer; EMP, glycolysis pathway; EPOX, cytochrome P-450 epoxygenase; FFCT, Fufangchangtai; GM, gut microbiota; HMDB, Human Metabolome Database; HPLC, high-performance liquid chromatography; IARS2, isoleucyl-tRNA synthetase 2; KRS, lysyl-tRNA synthetase; LC/MS, Liquid chromatography–mass spectrometry; LOX, lipoxygenase; OTUs, operational taxonomic units; PPP, pentose phosphate pathway; PCoA, principal coordinates analysis; QC, quality control; ROS, reactive oxygen species; SPF, specific pathogen-free; TCA, tricarboxylic acid cycle; TCM, traditional Chinese medicine.
