# Supplementary figures and images for: Disordered Gut Microbiota in Colorectal Tumor-Bearing Mice Altered Serum Metabolome Related to Fufangchangtai
Source: Front Pharmacol. 2022 May 26;13:889181. doi: 10.3389/fphar.2022.889181 (PMC9178095; doi:10.3389/fphar.2022.889181)

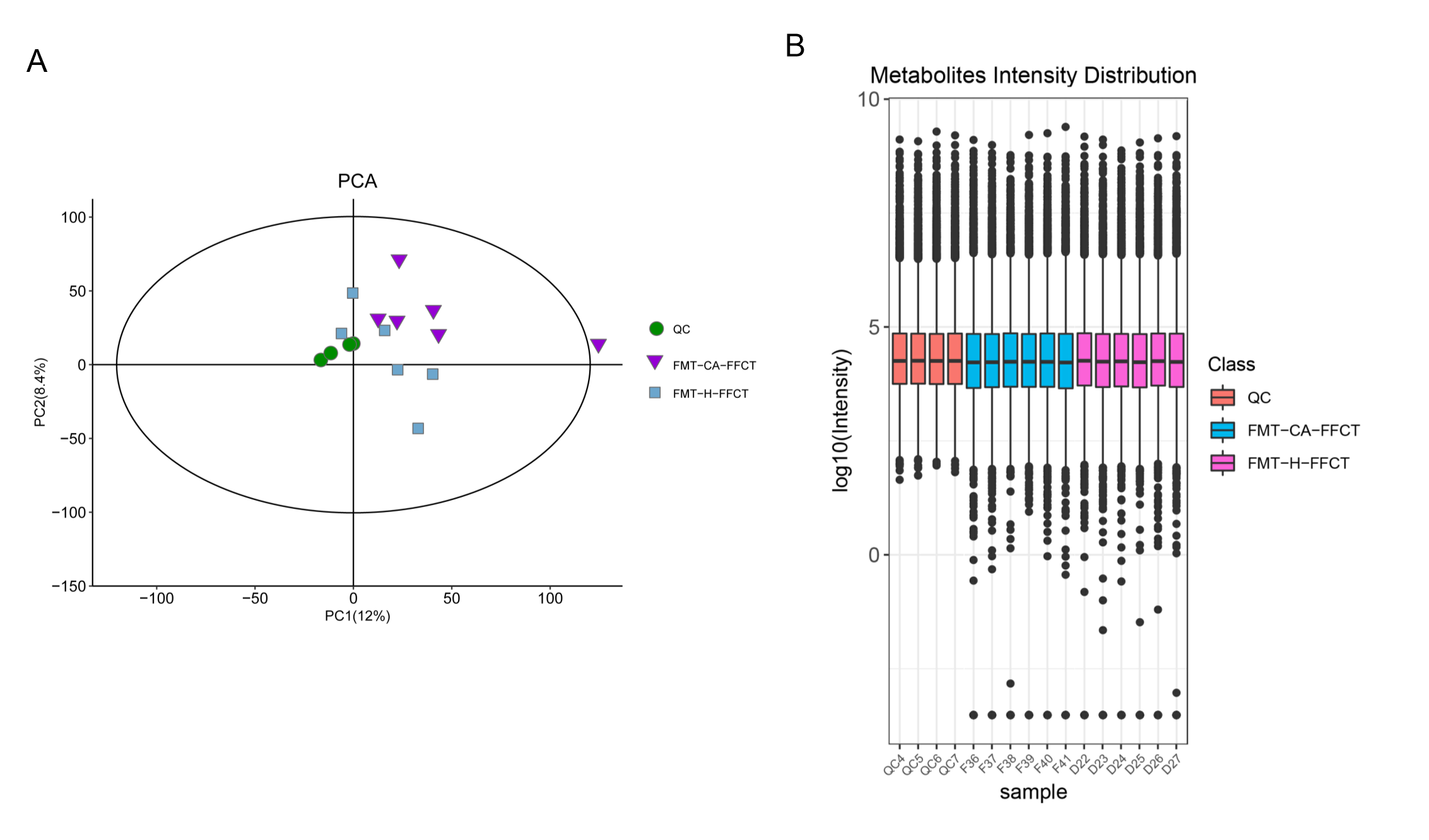

Supplement: Supplementary file 1 [file Image6.TIF]

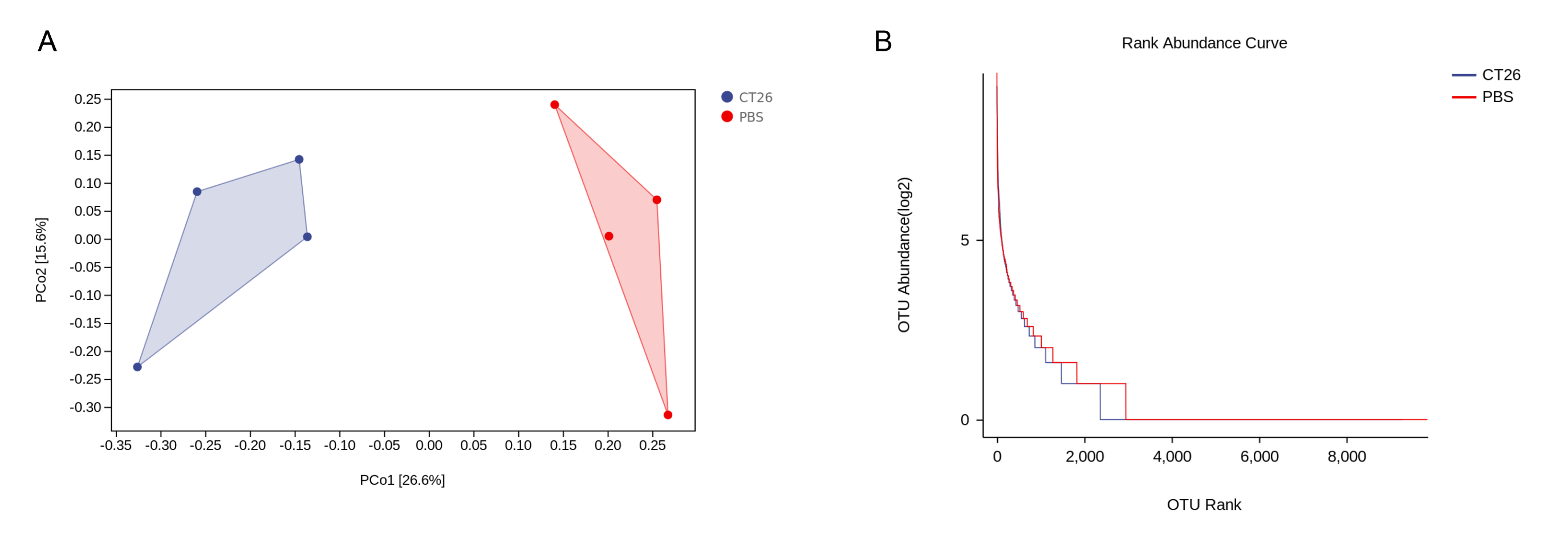

Supplement: Supplementary file 3 [file Image3.TIF]

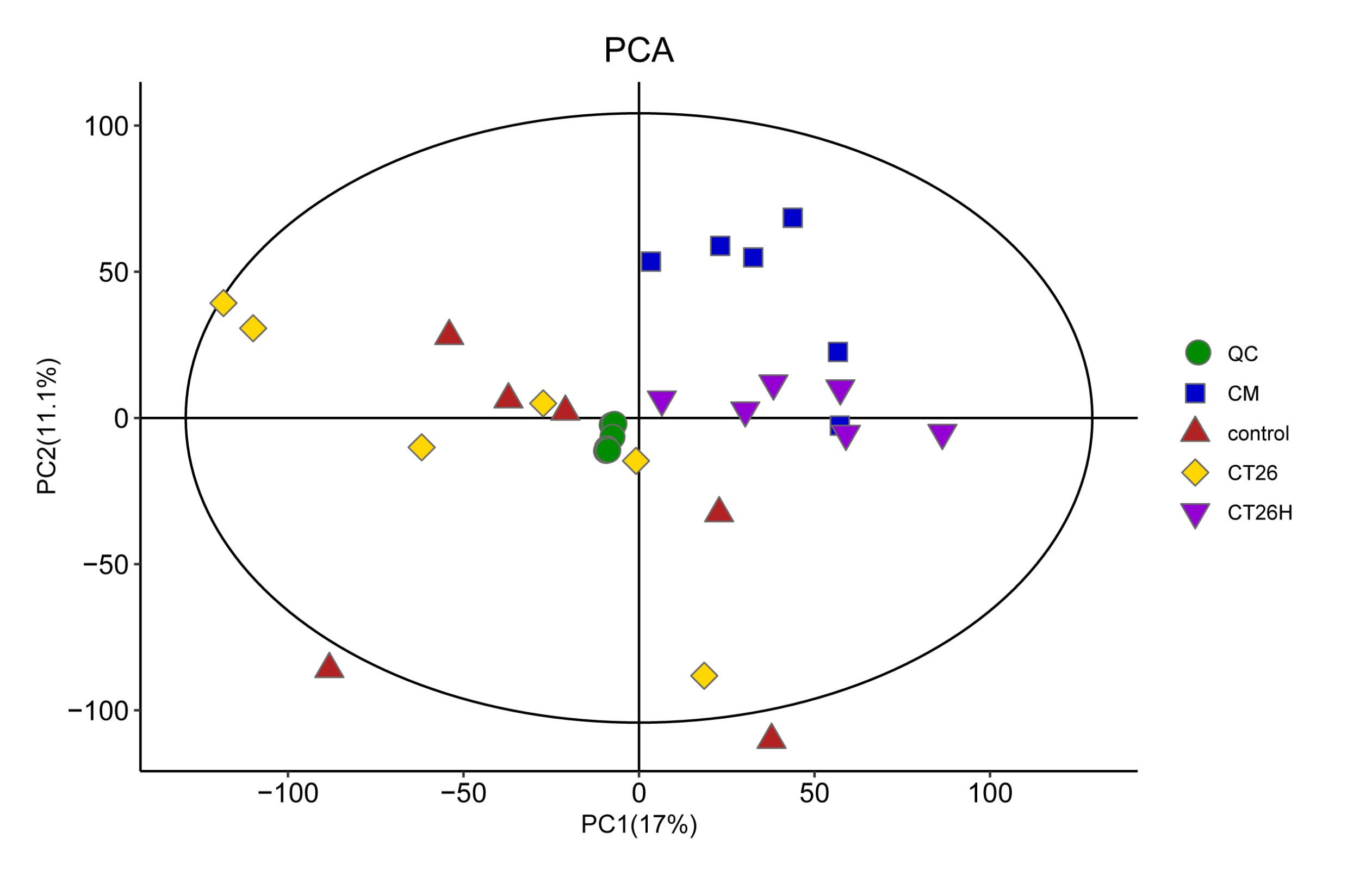

Supplement: Supplementary file 4 [file Image4.TIF]

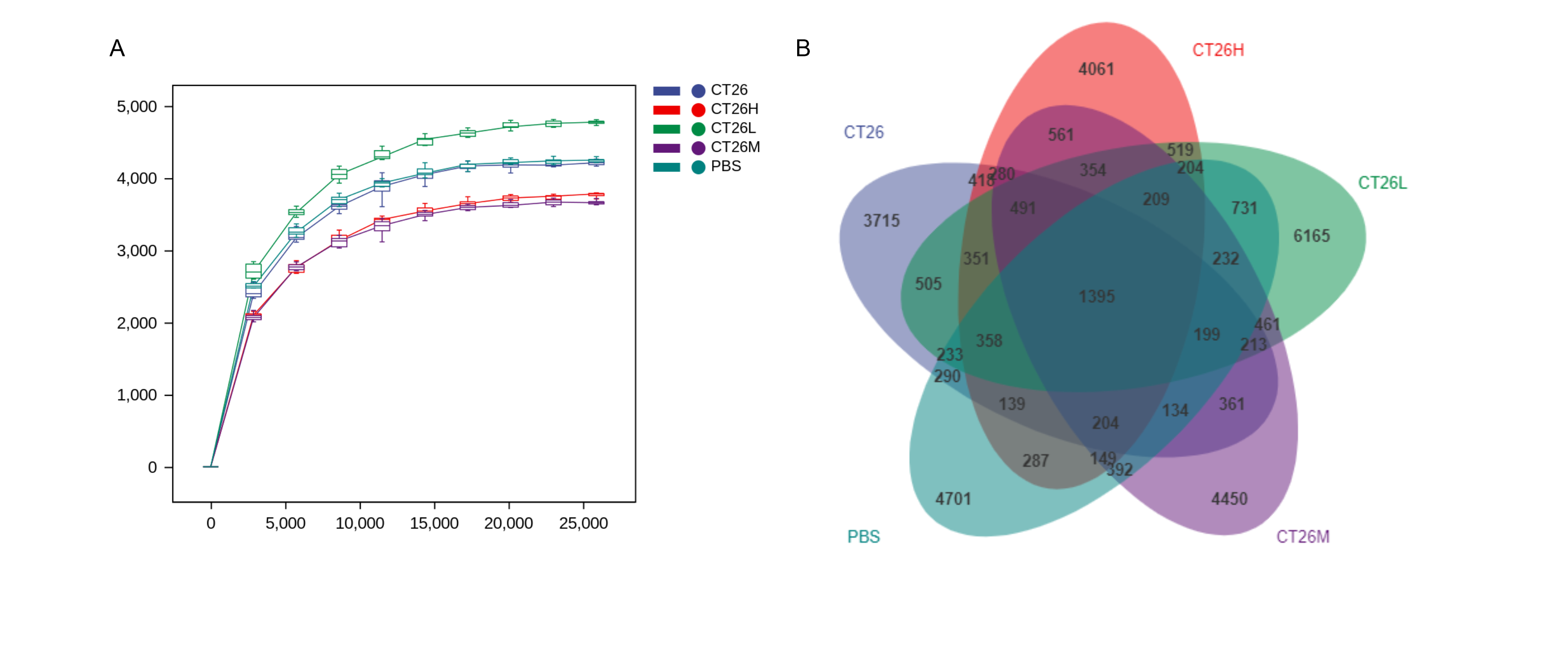

Supplement: Supplementary file 5 [file Image2.TIF]

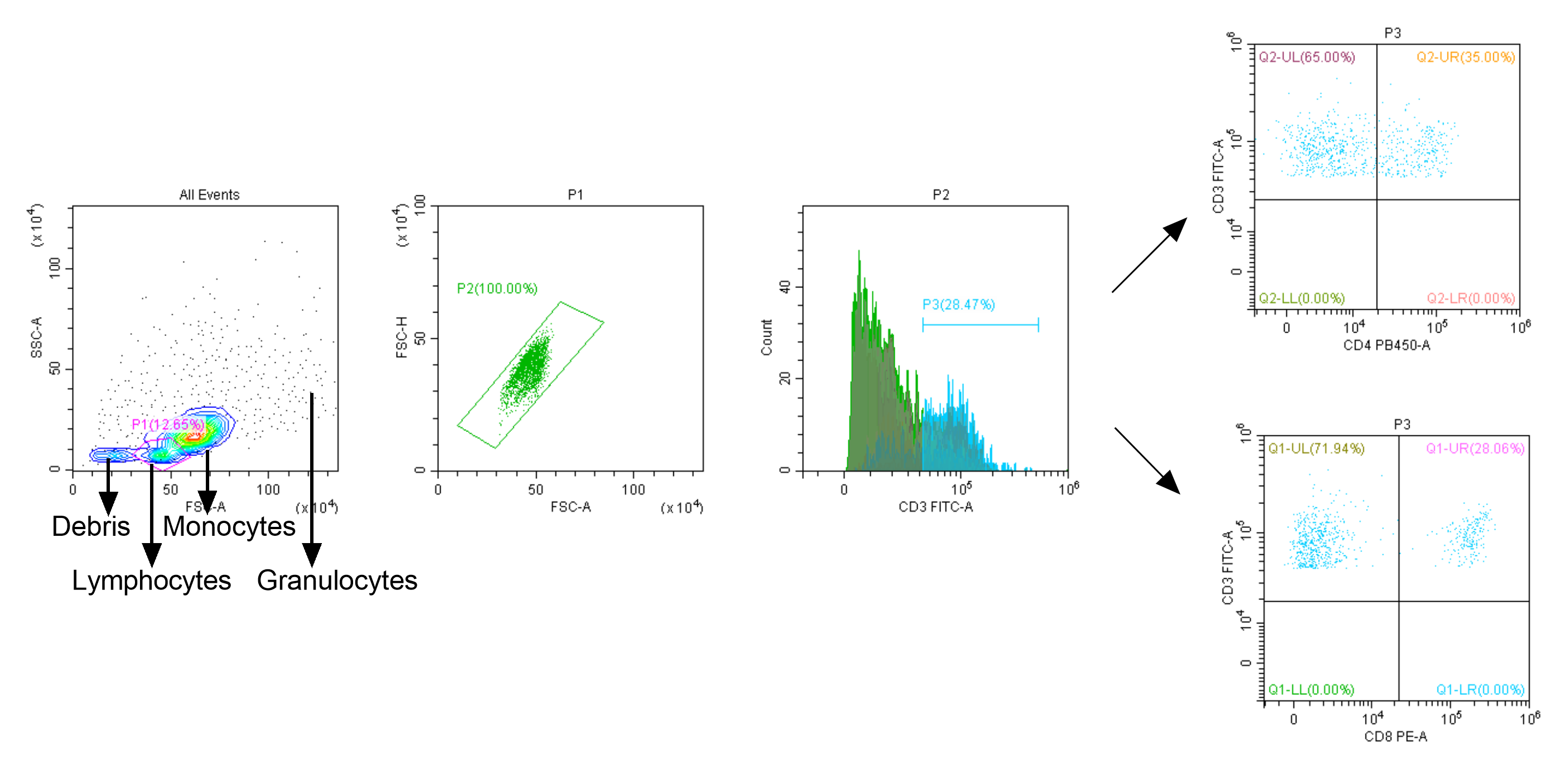

Supplement: Supplementary file 6 [file Image1.TIF]

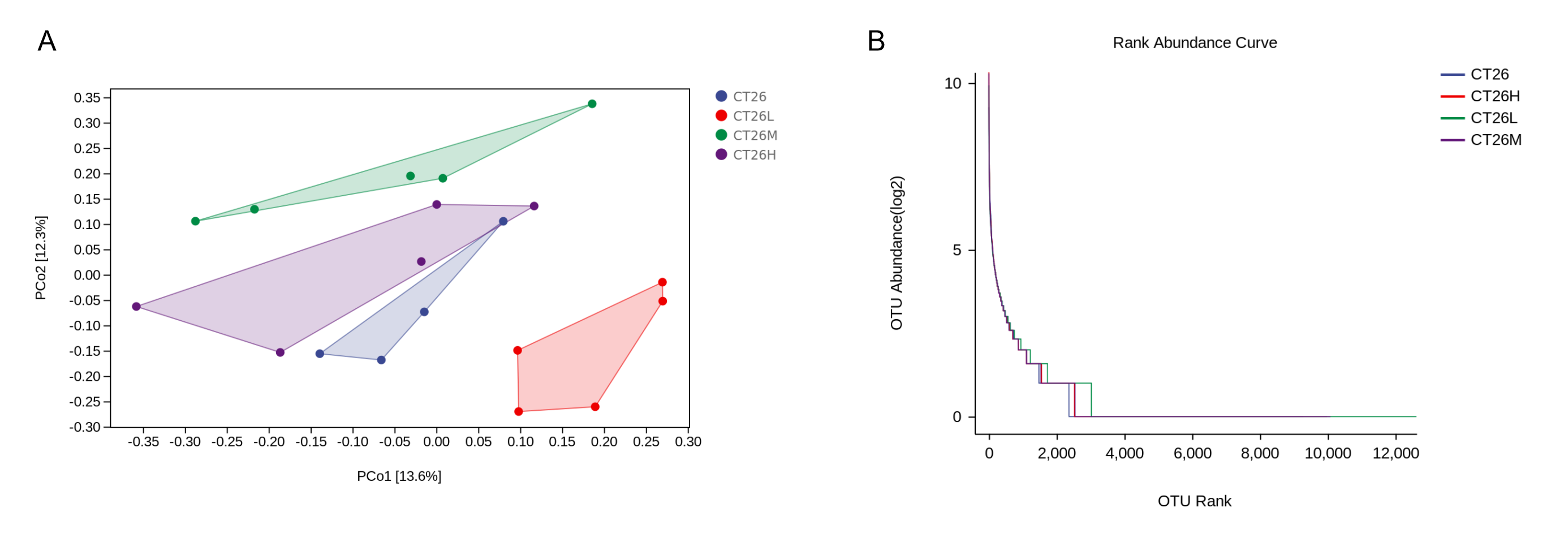

Supplement: Supplementary file 7 [file Image7.TIF]

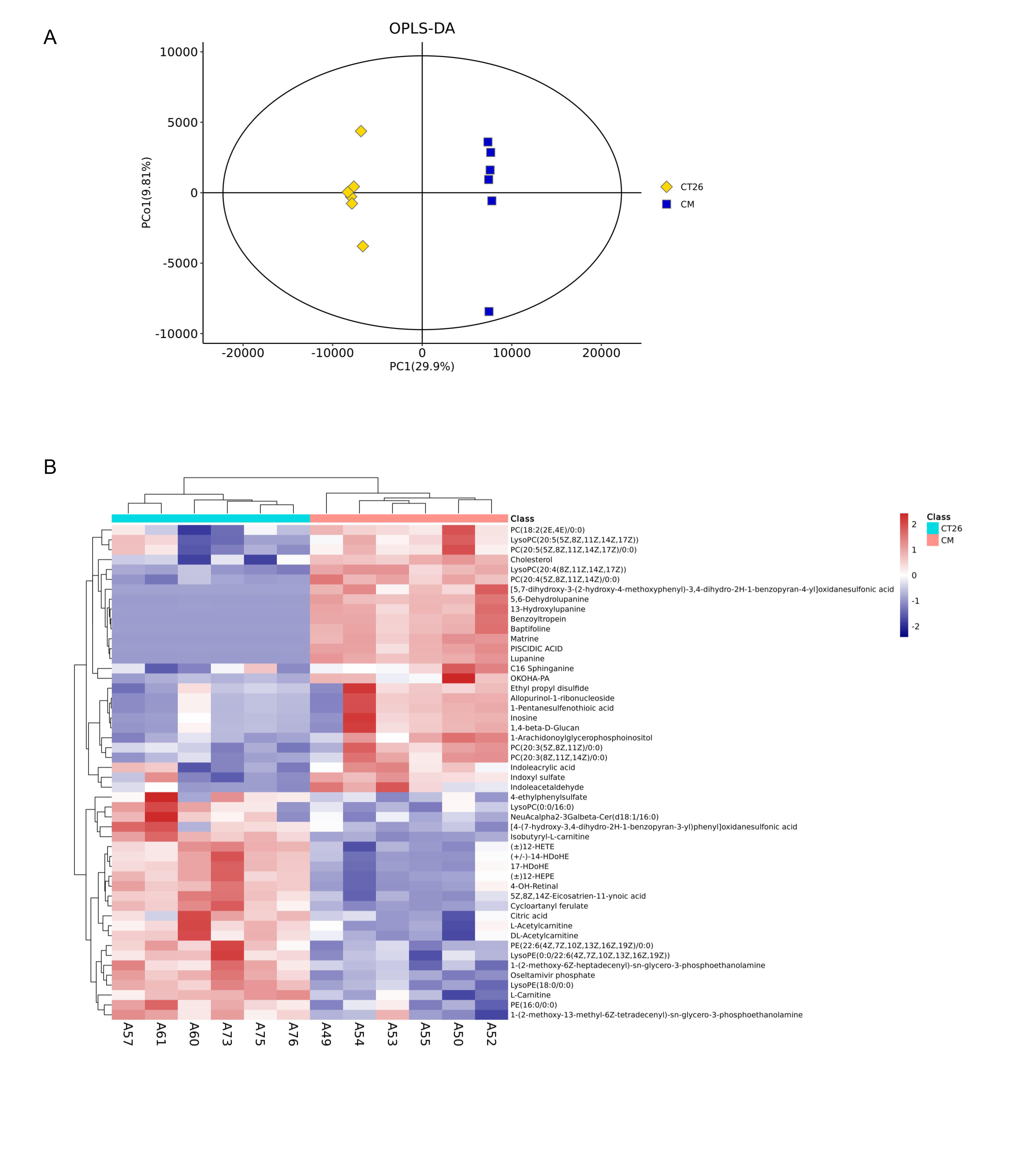

Supplement: Supplementary file 8 [file Image5.TIF]
