# Supplementary material for: Disordered Gut Microbiota in Colorectal Tumor-Bearing Mice Altered Serum Metabolome Related to Fufangchangtai
Source: Front Pharmacol. 2022 May 26;13:889181. doi: 10.3389/fphar.2022.889181 (PMC9178095; doi:10.3389/fphar.2022.889181)
Supplement: Supplementary file 2 [file Table1.DOCX]

Supplemental Table 1

General information of fecal-providing patients in FMT experiment

| No. | Gender | Age(years) | Stage |
| --- | --- | --- | --- |
| CRC-1 | male | 70 | Ⅲ |
| CRC-2 | female | 34 | Ⅲ |
| CRC-3 | female | 54 | Ⅲ |
| CRC-4 | female | 65 | Ⅲ |
| H1 | female | 62 | - |
| H2 | male | 51 | - |
| H3 | female | 56 | - |
| H4 | female | 48 | - |
